# Maternal Thyroid Hormone Replacement Therapy Exposure and Language and Communication Skills of Offspring at 8 Years of Age

**DOI:** 10.1001/jamanetworkopen.2019.12424

**Published:** 2019-10-02

**Authors:** Anna S. Frank, Angela Lupattelli, Ragnhild E. Brandlistuen, Hedvig Nordeng

**Affiliations:** 1 Pharmacoepidemiology and Drug Safety Research Group, Department of Pharmacy, University of Oslo, Oslo, Norway; 2 Department of Child Health and Development, Norwegian Institute of Public Health, Oslo, Norway

## Abstract

**Importance:**

Hypothyroidism during pregnancy is associated with neurodevelopmental delays in the offspring. However, it remains unknown whether prenatal thyroid hormone replacement therapy (THRT) has benefits regarding children’s language and communication skills.

**Objective:**

To quantify associations between prenatal THRT exposure and risk of language impairment diagnosis and parent-reported symptoms of language and communication skill deficits in offspring at 8 years of age.

**Design, Setting, and Participants:**

The Norwegian Mother, Father and Child Cohort Study (MoBa), a nationwide population-based cohort study, recruited pregnant women from throughout Norway between June 1999 and December 2008. MoBa was linked to several nationwide registries: the Norwegian Medical Birth Registry, Norwegian Prescription Database, and Norwegian Patient Registry. For this study, the analyzed cohort was restricted to singleton pregnancies resulting in a live-born infant, enrolled in the MoBa between 2005 and 2008. Statistical analysis was performed from January 2 to May 7, 2019.

**Exposures:**

In both study samples, mother-child pairs were categorized into 3 mutually exclusive groups: THRT exposure during pregnancy, based on dispensed prescription records; unexposed to THRT during pregnancy (population comparison); and mothers initiating THRT after delivery (THRT after delivery), comprising incident postpartum THRT users.

**Main Outcomes and Measures:**

Two defined study samples were analyzed with different outcome measures. In the Norwegian Patient Registry sample, outcome was defined by a diagnosis of language and speech impairment. In the MoBa sample, children were followed up until age 8 years via parental self-completed questionnaires. Hazard ratios were calculated for language impairment diagnosis, estimated by Cox proportional hazards regression. Standardized mean score (β) was calculated for parent-reported symptoms of language and communication deficits, estimated using generalized linear models.

**Results:**

The Norwegian Patient Registry sample included 53 862 mother-child pairs (mean [SD] age, 30.4 [4.6] years; offspring, 26 145 girls and 27 717 boys; 1204 pairs exposed to THRT [2.2%]) and the MoBa sample included 23 686 mother-child pairs (mean [SD] age, 30.8 [4.4] years; offspring, 11 536 girls and 12 150 boys; 532 pairs exposed to THRT [2.2%]). Language and speech impairment diagnosis was not significantly associated with prenatal THRT exposure compared with the unexposed group (adjusted hazard ratio, 0.75; 95% CI, 0.38-1.43) or the THRT after delivery group (adjusted hazard ratio, 0.63; 95% CI, 0.26-1.53). Language outcomes also did not significantly differ between these groups.

**Conclusions and Relevance:**

There was no significant difference in child outcomes between children exposed to THRT in the prenatal period compared with children in the population comparison group. These results support current guidelines recommending hypothyroidism treatment during pregnancy. Future research should further examine the use of THRT after delivery or a proper disease comparison group.

## Key Points

**Question:**

Is prenatal exposure to thyroid hormone replacement therapy associated with children’s language, speech, and communication skills at 8 years of age?

**Findings:**

In this nationwide population-based cohort linkage study of 53 862 mother-child pairs in Norway, prenatal thyroid hormone replacement therapy exposure was not associated with diagnoses and symptoms of child language and speech impairment, compared with the group not exposed to thyroid hormone replacement therapy or the group that received thyroid hormone replacement therapy after delivery.

**Meaning:**

These findings indicate that the children of mothers treated with thyroid hormone replacement therapy for hypothyroidism were not associated with symptoms of or increased risk of language impairment.

## Teaser

This population-based cohort study examines associations between prenatal thyroid hormone replacement therapy exposure and risk of language impairment and parent-reported symptoms of language and communication skill deficits in offspring at 8 years of age.

## Introduction

Maternal thyroid hormones are essential for the offspring’s normal brain development, including dendritic and axonal growth, synaptogenesis, neuronal development, and myelination.^1^ A lack of thyroid hormones during gestation can cause neurodevelopmental delays in the offspring.^2^ Therefore, it is recommended that women with hypothyroidism receive thyroid hormone replacement therapy (THRT) during pregnancy.^3^ However, few studies have investigated whether in utero THRT exposure influences language, speech, and communication skills in children in a real-world setting.

One recent study based on 2 randomized placebo-controlled trials reported no improvement in IQ levels, language development, or motor development among the 5-year-old children of mothers with hypothyroidism or subclinical hypothyroidism who received prenatal THRT.^4^ Similarly, Lazarus et al^5^ reported that antenatal screening and consequent THRT use did not improve cognitive function among the 3-year-old children of mothers with hypothyroidism. Caveats of these studies include relatively small sample sizes and late treatment onset, often starting after gestational week 8.^4^ Therefore, it remains unclear whether THRT exposure in utero can prevent language problems.

In the present nationwide cohort study, our primary aim was to analyze whether prenatal THRT exposure was associated with less frequent diagnosis of language and speech impairment in 8-year-old children. Our secondary aim was to examine the association of prenatal THRT exposure with parent-reported language, speech, and communication skill deficits at this age. We hypothesized that children born to women who received prenatal THRT would have an equal or lower risk of language and communication skill problems compared with children born to women without hypothyroidism or with incident postnatal hypothyroidism, respectively.

## Methods

### Data Sources

All legal residents of Norway are given a unique 11-digit personal identification number. Using this number, we combined information from the following data sources. The Norwegian Mother, Father and Child Cohort Study (MoBa) is a prospective population-based cohort study of pregnancies in Norway, which was initiated in 1999 by the Norwegian Institute of Public Health and involves ongoing follow-up.^6^ From June 1999 to December 2008, all pregnant women in Norway were invited to participate via a postal invitation sent in relation to the routine ultrasound examination around gestational week 17. Approximately 41% of the invited women consented to participate, and completed a questionnaire (MoBa Q1). These women were followed up via a series of questionnaires to be completed at gestational weeks 22 (MoBa Q2) and 30 (MoBa Q3); at 6 (MoBa Q4) and 18 (MoBa Q5) months after delivery; and at 3 (MoBa Q6), 5 (MoBa Q5-y), 7 (MoBa Q7-y), and 8 (MoBa Q8-y) years after delivery.^7^ Child follow-up is still ongoing. Compared with the general Norwegian population, MoBa participants were generally older, of higher socioeconomic status, less often smokers, and more commonly adhered with folic acid and other nutritional recommendations.^8^ Prospective fathers also completed a questionnaire at gestational week 16. The MoBa cohort now includes 114 500 children, 95 200 mothers, and 75 200 fathers.^8^ Our present study was based on version 10 of the quality-assured data released for research purposes in 2017. This study followed the Strengthening the Reporting of Observational Studies in Epidemiology (STROBE) reporting guideline for cohort studies.^13^ The establishment of MoBa and subsequent data collection were previously based on a license from the Norwegian Data Protection Agency and approval from The Regional Committee for Medical Research Ethics and are now based on regulations related to the Norwegian Health Registry Act. The overall MoBa study was approved by the Norwegian Data Inspectorate and the Regional Committee for Medical Research Ethics. The current study was approved by the Regional Committee for Medical Research Ethics. All participants provided written informed consent prior to participation.

The Medical Birth Registry of Norway (MBRN) is a nationwide health registry containing information about all births in Norway.^9^ This registry includes confirmed medical records related to maternal health before and during pregnancy.^9^

The Norwegian Prescription Database is a nationwide prescription registry established in January 2004. Since then, all pharmacies in Norway have been obligated to electronically send the Norwegian Institute of Public Health data regarding all prescribed drugs dispensed to individuals in ambulatory care.^10^

The Norwegian Patient Registry (NPR) records individual patient diagnoses according to *International Classification of Diseases and Related Health Problems, Tenth Revision* (*ICD-10*) codes. Since 2008, all government-owned and government-financed hospitals and outpatient clinics have mandatorily reported this information to receive financial reimbursement.^11,12^

### Study Samples

For this study, the analyzed cohort was restricted to singleton pregnancies resulting in a live-born infant, enrolled in the MoBa between 2005 and 2008. All included mothers completed the MoBa Q1 and MoBa Q3 and were successfully linked to data in the Norwegian Prescription Database. Mothers were excluded if the MBRN indicated that they had prescriptions for other thyroid disorders (Anatomical Therapeutic Chemical [ATC] code H03B, or a combination of ATC codes H03AA and H03B, and *ICD-10* code e0-other), incomplete information, or a hyperthyroidism diagnosis (*ICD-10* code e05).

This analytical cohort was divided into 2 study samples: the NPR sample and the MoBa sample. Both sample sets excluded mother-child pairs with conflicting information regarding thyroid diagnoses from the MBRN and NPR during gestation (17 of 53 969 [0.03%] in the MoBa study sample; and 47 of 53 969 [0.08%] in the NPR study sample) and those who received THRT only prior to but not during pregnancy. The final NPR study sample comprised 53 862 mother-child pairs and the final MoBa study sample comprised 23 686 mother-child pairs.

### Exposure

In both the NPR and MoBa study samples, we identified mother-child pairs with hypothyroidism based on dispensed THRT prescriptions. A previous study reported varying κ values for the agreement between maternal self-reporting and dispensed prescriptions during pregnancy.^14^ The κ in the whole gestational period was 0.91 (95% CI, 0.89-0.92) and ranged from 0.76 (95% CI, 0.76-0.77) for weeks 0 to 4 to 0.39 (95% CI, 0.38-0.40) for weeks 25 to 28. In addition, the proportion of self-reported THRT use was consistently lower than that of filled prescription records.

**Figure 1.**
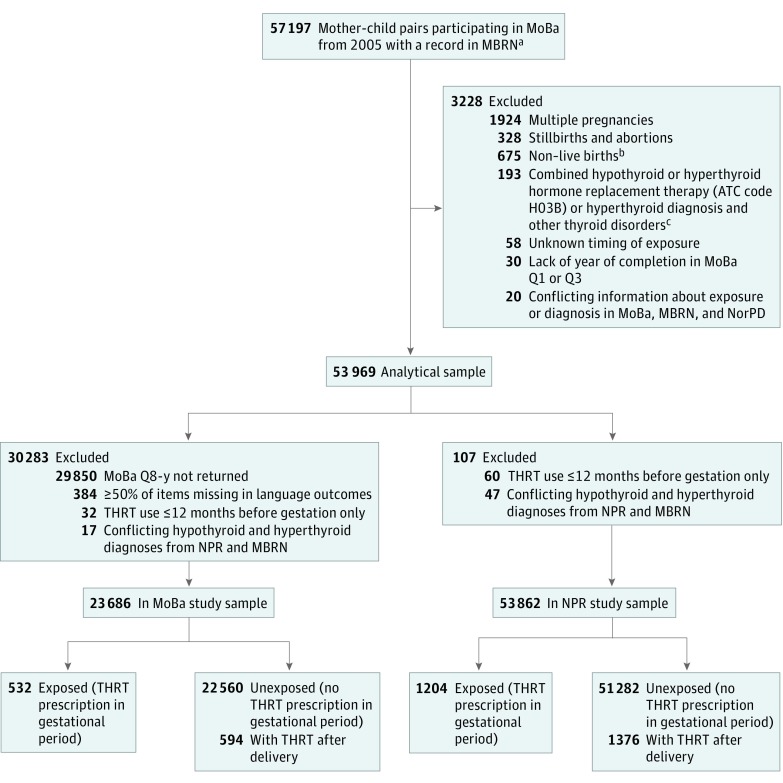
Flowchart of Study Samples ATC indicates Anatomical Therapeutic Chemical Classification System code; MBRN, Medical Birth Registry of Norway; MoBa, Norwegian Mother, Father and Child Cohort Study; MoBa Q1, MoBa questionnaire 1; MoBa Q3, MoBa questionnaire 3; MoBa Q8-y, MoBa questionnaire at child age 8 years; NorPD, Norwegian Prescription Database; NPR, Norwegian Patient Registry; and THRT, thyroid hormone replacement therapy. ^a^The NorPD was established in 2004, thus required restriction of the MoBa population to pregnancies recruited from 2005. ^b^Children who were born alive but died between birth and 2 years of age, emigrated, or had unknown follow-up status. ^c^Hyperthyroid diagnosis (*International Classification of Diseases and Related Health Problems, 10th Revision* [*ICD-10*] code e05), and other thyroid diagnosis (*ICD-10* code e0-other) from the MBRN.

The THRT-exposed group included mother-child pairs having at least 1 dispensed THRT prescription from the date of the last menstrual period to delivery, including 1204 pairs in the NPR sample and 532 pairs in the MoBa sample. We also identified 2 comparison groups in each study sample: an unexposed group and a group that received THRT after delivery. The unexposed (population comparison) group included mother-child pairs with no dispensed THRT prescription before or during gestation or after delivery (51 282 pairs in the NPR sample, and 22 560 in the MoBa sample). The THRT after delivery group included mother-child pairs who filled incident THRT prescriptions within 1 year after delivery (1376 pairs in the NPR sample, and 594 in the MoBa sample) (Figure 1). Exposure to THRT was classified based on the ATC Classification System and included thyroid hormones (ATC code H03AA).^15^

### Outcomes

We examined child language and communication skills from the perspectives of diagnosis and symptoms. Details regarding the choice of outcomes are described in eMethods 1 in the Supplement.

### Language Diagnosis

For the NPR sample, we retrieved information regarding language and speech impairment diagnosis (*ICD-10* code F80) up to approximately 8 years of age. Subdiagnoses included in the *ICD-10* code F80 are listed in eMethods 1 in the Supplement.

### Language and Communication Skill Outcomes

In the MoBa sample, parent-reported language and communication skill outcomes were measured when the child was approximately 8 years of age, via the MoBa Q8-y. These data were available for 38% of the MoBa population.^16^ The MoBa Q8-y applied the following widely used and validated psychometric instruments: the short Children’s Communication Checklist,^17^ the Language 20 Semantic subscale,^18^ and the Social and Communication Questionnaire,^19^ plus additional questions about child pronunciation skills. Whenever at least 50% of the questionnaire items were completed, we summed the item scores and calculated standardized mean scores.

The short Children’s Communication Checklist–2 includes 16 questions from the original Children’s Communication Checklist–2 developed by Norbury et al.^20^ These 16 items can be clustered into factors of strengths and difficulties. We reversed the items of the strengths scale to build the *Z*-score, such that a higher score was associated with lower skills. Semantic language problems were calculated based on 8 items of the Language 20 Semantic subscale.^21^ Autism symptoms were calculated based on all 40 items in the Social and Communication Questionnaire.^22^ The first 2 questions from the Child Pronunciation Skills questionnaire^23^ were used to measure pronunciation problems, and all 4 questions defined speech difficulties.

### Covariates

Covariates were selected based on previous findings and theoretically plausible confounders. From MoBa Q1 and MoBa Q3, we obtained information about sociodemographic characteristics, including educational level, income, body mass index at conception, smoking habits, and alcohol consumption. In MoBa Q1, lifetime history of major depression was measured by the lifetime major depression scale of Kendler et al.^24^ Maternal age, marital status, paternal age, sex of newborn, and parity were retrieved from the MBRN. Paternal educational level was retrieved from the MoBa father questionnaire. Perinatal use of folic acid supplements, alone or with additional supplements, was ascertained from MoBa Q1 and MoBa Q3. From the Food Frequency Questionnaire (MoBa Q2), we determined whether maternal fiber intake was above or below the median intake of the study population, which was used as a proxy for a healthy lifestyle.^25^

Somatic comorbidities were classified as medicated or nonmedicated, depending on whether the woman had a registered diagnosis in the MBRN, and reported on MoBa Q1 as receiving treatment for epilepsy (ATC code N03A), arthritis (ATC codes L04A, M01, and N02), type 1 and 2 diabetes and gestational diabetes (ATC codes A10A, A10B, and A10X), anemia (ATC codes B03A, B03B, and B03X), or cardiovascular disorders (ATC codes C01-C10). Mental comorbidities (depression and/or anxiety) were determined from MoBa Q1 and MoBa Q3, and categorized as medicated or nonmedicated, depending on whether the woman reported psychotropic drug use (ATC codes N05 and N06). When the child was 8 years of age, we calculated maternal symptoms of anxiety and depression using the Hopkins Symptoms Checklist.^26^ When the child was 5 years of age, we used the MoBa Q-5y to collect information regarding multilingualism and family history of language, reading, and writing problems. Data regarding maternal hypothyroid diagnoses were available from the NPR and MBRN.^11^

### Statistical Analysis

Statistical analysis was performed from January 2 to May 7, 2019. For both study samples, we summarized and described maternal and child characteristics for each of the 3 exposure groups. To calculate the hazard ratio and 95% CI, we used Cox proportional hazard regression models and compared the risk of language and speech impairment diagnosis after THRT exposure vs the 2 comparison groups. We calculated the standardized mean score (β) and 95% CI using generalized linear regression (Gaussian family, identity link function) and estimating the differences in parent-reported outcomes between the 3 exposure groups. These analyses were performed with adjustment for maternal age, educational level, income, smoking and alcohol habits during gestation, parity, lifetime history of major depression, body mass index at conception, folic acid and other supplement use, and mental and somatic comorbidity variables. Because there were few diagnosis events in the NPR study sample, we adjusted only for maternal income and perinatal use of folic acid and other supplements, to avoid overfitting when considering the THRT after delivery group.

#### Handling of Missing Data

In the NPR study sample, 24.9% of mother-child pairs had missing information in 1 or several covariates; in the MoBa study samples, 41.5% of pairs had missing information in 1 or several covariates. Missing covariate values were imputed using multiple imputation by chained equations based on the missing at random assumption.^27^ We generated 10 imputed sets based on variables included in the final statistical model (eg, outcomes, exposure, covariates, and other auxiliary variables) (eTable 1 in the Supplement).^28,29^ In both analyses, we accounted for sibling clusters. The results of all imputed sets were pooled according to Rubin’s Rules.^30^ We tested the Cox proportional hazard regression assumption based on scaled Schoenfeld residuals, revealing that the assumption was not met for medicated mental comorbidity or high income levels. However, globally, we found no association of the model with time.^31^

#### Sensitivity Analyses

We performed several preplanned sensitivity analyses to check the robustness of our findings. First, we adjusted for additional covariates, including paternal variables and child sex in both samples, as well as maternal depression, multilingualism, and family history of reading, writing, and language problems in the MoBa sample. Complete case analysis was also performed. Furthermore, we restricted the analysis to women with a medically confirmed hypothyroid diagnosis and to women with consistent THRT use (defined by dispensed prescriptions in all trimesters, similar to Turunen et al^32^). Finally, in the MoBa sample, we split the THRT exposure group into disjoint medication patterns using group-based trajectory models, as previously described in detail.^14,33^ Our rationale for these sensitivity analyses is provided in eMethods 2 in the Supplement. Power calculations for the NPR and MoBa study samples are presented in eMethods 3 in the Supplement.

Statistical analyses were performed in R, version 3.4.4 (R Project for Statistical Computing). Multiple imputation was performed using the “mice” package^27^ and regression analysis using the “survey” package.^34^ The final estimates were pooled using the “mitools” R package.^35^ Group-based trajectory models were built using the “traj” Stata plugin (Stata, version 15.1 [StataCorp]).^36^ Standardized differences were calculated based on R package “tableone.”^37^ Statistical significance was set to a 2-sided *P* < .05.

## Results

In the NPR study sample, 1204 mother-child pairs (2.2%) were exposed to THRT during pregnancy, 1376 (2.6%) started THRT after delivery, and 51 282 (95.2%) were unexposed during pregnancy (Figure 1). In the MoBa study sample, 532 mother-child pairs (2.2%) were exposed to THRT during pregnancy, 594 (2.5%) started THRT after delivery, and 22 560 (95.2%) were unexposed during pregnancy. Overall, relative to the other comparison groups, mothers receiving prenatal THRT were older, were more often obese, and more commonly experienced comorbidities.

Table 1 presents maternal characteristics for the NPR study sample. Compared with the NPR sample, mothers in the MoBa study sample (eTable 2 in the Supplement) were less commonly smokers, had higher socioeconomic and educational levels, and were generally healthier. These characteristics have been previously reported for the MoBa study.^8^ During follow-up, the cumulative incidence of language and speech impairment diagnosis in children was 0.17% among the group exposed to THRT, 0.07% among the group not exposed to THRT, and 0.13% among children of mothers initiating THRT after delivery. The median ages at first diagnosis were 6.4 years among the group exposed to THRT, 6.2 years among the group not exposed to THRT, and 5.1 years among children of mothers initiating THRT after delivery. The risk of a language and speech impairment diagnosis was lower among children whose mothers received prenatal THRT, compared with unexposed women (adjusted hazard ratio, 0.75; 95% CI, 0.38-1.43) and the THRT after delivery group (adjusted hazard ratio, 0.63; 95% CI, 0.26-1.53), although the 95% CI crossed the null for both estimates (Table 2). Children in the THRT after delivery group received a diagnosis approximately 1 year earlier than in the THRT exposed and unexposed groups (5.1 vs 6.4 vs 6.2 years) (Table 2).

**Table 1.** Characteristics of the Norwegian Patient Registry Sample^a^

Variables	Mother-Child Pairs, No. (%)			Standardized Difference of THRT	
	THRT Exposed (n = 1204)	Unexposed (n = 51 282)	THRT After Delivery (n = 1376)	Exposed vs Unexposed	Exposed vs THRT After Delivery
Maternal age, y					
≤24	68 (5.6)	5327 (10.4)	137 (9.9)	0.272	0.212
25-29	325 (26.9)	16 388 (31.9)	413 (30.0)		
30-34	479 (39.8)	20 104 (39.2)	536 (38.9)		
≥35	332 (27.6)	9463 (18.4)	290 (21.1)		
Paternal age, y					
≤24	32 (2.6)	2331 (4.5)	60 (4.4)	0.216	0.151
25-29	205 (17.0)	11 202 (21.8)	286 (13.5)		
30-34	432 (35.8)	19 821 (38.7)	494 (35.9)		
≥35	529 (43.9)	17 780 (34.7)	532 (38.7)		
BMI at conception					
≤18	25 (2.1)	1609 (3.1)	31 (2.3)	0.280	0.088
19-24	617 (51.2)	31 476 (61.4)	747 (54.3)		
25-29	314 (26.0)	12 093 (23.6)	361 (26.2)		
≥30	214 (17.8)	4886 (9.5)	203 (14.7)		
Married or cohabiting					
Yes	1145 (95.1)	49 029 (95.6)	1302 (94.6)	0.024	0.022
No	59 (4.9)	2253 (4.4)	74 (5.3)		
Parity					
Multiparity	695 (57.7)	26 935 (52.5)	747 (54.3)	0.105	0.069
Primiparity	509 (42.3)	24 347 (47.5)	629 (45.7)		
Maternal educational level (ongoing), y					
<9	18 (1.5)	831 (1.6)	31 (2.2)	0.011	0.115
9-12	299 (24.8)	12 813 (24.9)	398 (28.9)		
13-16	506 (42.3)	21 413 (41.7)	532 (38.6)		
>16	359 (29.8)	15 267 (29.8)	390 (28.3)		
Paternal educational level (ongoing), y					
<9	43 (3.6)	1761 (3.4)	64 (4.6)	0.031	0.128
9-12	435 (36.1)	18 401 (35.9)	562 (40.8)		
13-16	321 (26.7)	14 300 (27.9)	336 (24.4)		
>16	336 (27.9)	13 857 (27.0)	335 (24.3)		
Maternal income, $^b^					
<16 013	296 (24.5)	12 060 (23.5)	365 (26.5)	0.058	0.115
16 013-54 443	673 (55.8)	30 169 (58.8)	801 (58.2)		
>54 443	191 (15.9)	7409 (14.4)	166 (12.0)		
Smoking during pregnancy					
Yes	42 (3.5)	2841 (5.5)	107 (7.7)	0.102	0.203
No	923 (76.6)	39 172 (76.4)	998 (72.6)		
Stopped	74 (6.1)	3192 (6.2)	93 (6.7)		
Alcohol use during pregnancy^c^					
Yes	235 (19.5)	11 769 (22.9)	319 (23.1)	0.085	0.101
No	914 (75.9)	37 527 (73.2)	999 (72.6)		
LTHMD^d^					
Yes	430 (35.7)	11 948 (23.3)	433 (31.5)	0.273	0.095
No	754 (62.6)	38 036 (74.2)	905 (65.7)		
Mental comorbidity					
Medicated	64 (5.3)	1225 (2.4)	49 (3.6)	0.172	0.105
Nonmedicated	140 (11.6)	4892 (9.5)	191 (13.8)		
No	1000 (83.0)	45 165 (88.1)	1136 (82.6)		
Somatic comorbidity^e^					
Medicated	149 (12.4)	1961 (3.8)	89 (6.5)	0.396	0.268
Nonmedicated	151 (12.5)	3444 (6.7)	110 (7.9)		
No	904 (75.1)	45 877 (89.4)	1177 (85.5)		
Folic acid and other supplements^f^					
Yes	867 (72.0)	34 140 (66.6)	955 (69.4)	0.118	0.057
No	337 (27.9)	17 142 (33.4)	421 (30.5)		
Fiber intake, g/d					
≥29.8	569 (47.2)	23 660 (46.1)	641 (46.6)	0.022	0.014
<29.8	635 (52.7)	27 622 (53.8)	735 (53.4)		
Sex of child					
Boy	630 (52.3)	26 361 (51.4)	726 (52.8)	0.018	0.009
Girl	574 (47.7)	24 921 (48.6)	650 (47.2)		
Maternal thyroid diagnosis^g^					
Hypothyroidism (*ICD-10* code e03)					
Yes	827 (68.7)	0	0	2.079	2.079
No	377 (31.3)	51 284 (100.0)	1376 (100.0)		

Abbreviations: BMI, body mass index (calculated as weight in kilograms divided by height in meters squared); *ICD-10*, *International Classification of Diseases and Related Health Problems, 10th Revision*; LTHMD, lifetime history of major depression; THRT, thyroid hormone replacement therapy; USD, US dollars.

^a^ A total of 13 465 of 53 862 mother-child pairs (25.0%) were missing information in important confounders. Missing percentage in variables: maternal educational level, 1005 (1.9%); income, 1732 (3.2%); alcohol, 2099 (3.9%); LTHMD, 1356 (2.5%); smoking, 6520 (11.9%); paternal educational level, 3111 (5.8%); paternal age, 158 (0.3%); and BMI, 1286 (2.4%).

^b^ Women’s income status (USD/y); 1.00 Norwegian kroner = 0.13 USD.

^c^ Alcohol consumption; no indicates “less than once per month,” and yes, “once or more per month.”

^d^ Presence of LTHMD included depression with and without external reason.

^e^ Somatic comorbidity includes epilepsy, arthritis, anemia, type 1 and 2 diabetes and gestational diabetes, and cardiovascular disorders.

^f^ Other supplements included vitamins (B1, B2, B6, B12, C, D, niacin, pantothenic acid, biotin), ω-3 fatty acids, and minerals (calcium, copper, chromium, iodine, iron, magnesium, selenium, and zinc).

^g^ *ICD-10* code e03 from the Norwegian Patient Registry and the Medical Birth Registry of Norway. Thyroid diagnoses, from before and during gestation, are available only for a subsample of the study population, because reporting thyroid diagnoses is not mandatory in the Medical Birth Registry of Norway and information in the Norwegian Patient Registry is incomplete if women received a diagnosis before 2008.

**Table 2.** Crude and Adjusted Hazard Ratios for Language and Speech Impairment Diagnosis^a^

Mother-Child Pairs	No. (%) of Diagnosis Events/y	Child Age at First Diagnosis, Median, y	Maximum Follow-up Time, y	Hazard Ratio (95% CI)			
				Model 1		Model 2	
				Crude	Adjusted^b^	Crude	Adjusted^c^
Unexposed (n = 51 282)	279 (0.07)	6.2	10.5	1 [Reference]	1 [Reference]	NA	NA
THRT after delivery (n = 1376)	11 (0.13)	5.1	8.2	NA	NA	1 [Reference]	1 [Reference]
THRT exposed (n = 1204)	10 (0.17)	6.4	9.7	0.95 (0.55-1.62)	0.75 (0.38-1.43)	0.52 (0.22-1.23)	0.63 (0.26-1.53)

Abbreviations: NA, not applicable; THRT, thyroid hormone replacement therapy.

^a^ Norwegian Patient Registry sample (n = 53 862).

^b^ All outcomes were adjusted for maternal age and educational level, income, parity, body mass index at conception, use of folic acid and other supplements, lifetime history of major depression, comedication for somatic and mental comorbidities, and smoking and alcohol use during pregnancy.

^c^ Adjusted for maternal income and use of folic acid and other supplements.

In the MoBa study sample, comparison between THRT-exposed and THRT-unexposed mother-child pairs revealed standardized mean differences ranging from −0.01 (95% CI, −0.10 to 0.08) for pronunciation problems to 0.05 (95% CI, −0.03 to 0.13) for autism symptoms. Similar mean differences were found when comparing THRT-exposed mother-child pairs with the THRT after delivery group (Table 3).

**Table 3.** Crude and Adjusted Standardized β Scores for Parent-Reported Language and Communication Skill Outcomes^a^

Mother-Child Pairs	Standardized β Score (95% CI)^b^									
	CCC-2		L-20		SCQ		Pronunciation Problems		Speech Difficulty	
	Unexposed	THRT After Delivery	Unexposed	THRT After Delivery	Unexposed	THRT After Delivery	Unexposed	THRT After Delivery	Unexposed	THRT After Delivery
Unexposed (n = 22 560)^c^	0 [Reference]	NA	0 [Reference]	NA	0 [Reference]	NA	0 [Reference]	NA	0 [Reference]	NA
THRT after delivery (n = 594)^d^	NA	0 [Reference]	NA	0 [Reference]	NA	0 [Reference]	NA	0 [Reference]	NA	0 [Reference]
THRT exposed (n = 532)	0.02 (−0.07 to 0.11)	0.05 (−0.08 to 0.17)	0.00 (−0.09 to 0.08)	0.03 (−0.09 to 0.15)	0.05 (−0.03 to 0.13)	0.07 (−0.05 to 0.19)	−0.01 (−0.10 to 0.08)	−0.04 (−0.16 to 0.09)	0.01 (−0.08 to 0.10)	0.02 (−0.11 to 0.14)

Abbreviations: β, standardized mean score difference; CCC-2, Children’s Communication Checklist–2; L-20, Language 20 Semantic subscale; NA, not applicable; SCQ, Social and Communication Questionnaire; THRT, thyroid hormone replacement therapy.

^a^ The Norwegian Mother, Father and Child Cohort Study sample (n = 23 686).

^b^ All outcomes were adjusted for maternal age and educational level, income, parity, body mass index at conception, use of folic acid and other supplements, lifetime history of major depression, comedication for somatic and mental comorbidities, and smoking and alcohol use during pregnancy.

^c^ Crude estimates: CCC-2 (β, 0.04; 95% CI, −0.04 to 0.13), L-20 (β, 0.01; 95% CI, −0.08 to 0.10), SCQ (β, 0.07; 95% CI, −0.02 to 0.15), pronunciation problems (β, 0.00; 95% CI, −0.08 to 0.09), and speech difficulty (β, 0.02; 95% CI, −0.07 to 0.11).

^d^ Crude estimates: CCC-2 (β, 0.03; 95% CI, −0.08 to 0.16), L-20 (β, 0.02; 95% CI, −0.10 to 0.15), SCQ (β, 0.07; 95% CI, −0.05 to 0.18), pronunciation problems (β, −0.04; 95% CI, −0.16 to 0.09), and speech difficulty (β, 0.01; 95% CI, −0.11 to 0.14).

### Sensitivity Analyses

**Figure 2.**
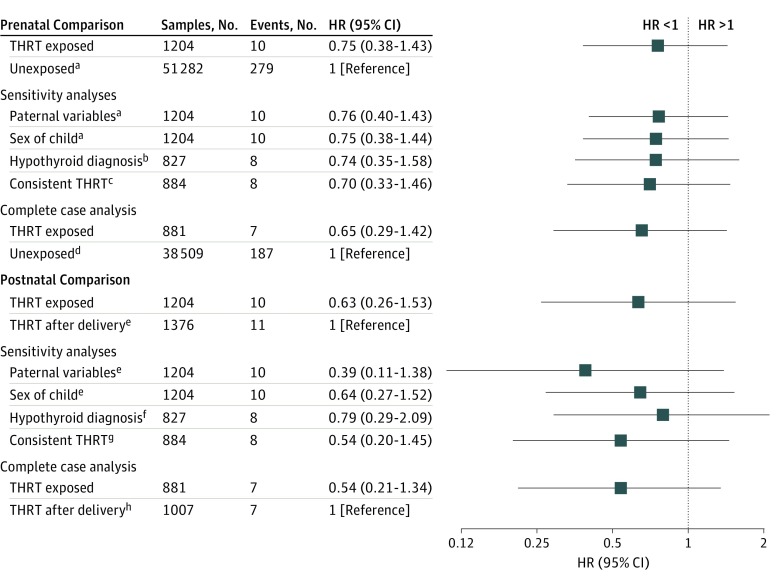
Main and Sensitivity Analyses in the Norwegian Patient Registry Study Sample HR indicates hazard ratio; THRT, thyroid hormone replacement therapy. ^a^Crude effect estimates HR, 0.95; 95% CI, 0.55-1.62. ^b^Crude effect estimates HR, 0.93; 95% CI, 0.51-1.69. ^c^Crude effect estimates HR, 1.14; 95% CI, 0.49-1.56. ^d^Crude effect estimates HR, 1.05; 95% CI, 0.64-1.71. ^e^Crude effect estimates HR, 0.52; 95% CI, 0.22-1.23. ^f^Crude effect estimates HR, 0.56; 95% CI, 0.22-1.42. ^g^Crude effect estimates HR, 0.42; 95% CI, 0.15-1.14. ^h^Crude effect estimates HR, 0.45; 95% CI, 0.16-1.28.

Complete case analysis in the MoBa study population showed fewer symptoms of pronunciation skills in the THRT-exposed group compared with the THRT unexposed group (β, −0.07; 95% CI, −0.15 to 0.00) and the THRT after delivery group (β, −0.13; 95% CI, −0.18 to 0.01). No difference was observed between the other sensitivity analyses and the main analysis (Figure 2; eTable 3, eTable 4, and eFigure in the Supplement).

Power calculations in the NPR study population showed that, for the THRT exposed group compared with the THRT unexposed group, a 67% reduced risk can be detected. For the THRT exposed group compared with the THRT after delivery group, an 86% reduced risk is detected. Power calculations in the MoBa study population showed that small effect sizes (Cohen *d*) could be detected. For the THRT exposed group compared with the THRT unexposed group, Cohen d was 0.13; for the THRT exposed group compared with the THRT after delivery group, Cohen *d* was 0.18.

## Discussion

To our knowledge, this was among the first studies to investigate the association between prenatal THRT exposure and language, speech, and communication skills in 8-year-old children. Our study is unique because it included both a medical diagnosis and parent-reported symptoms as child language developmental outcomes. The present findings suggest that children whose mothers received prenatal THRT had similar risks of language impairment in terms of diagnosis or symptoms as the population comparisons. These results are consistent with those of a previous Danish study^38^ that revealed that the risk of autism diagnosis did not differ between THRT-exposed mother-child pairs and population comparisons. This finding may suggest that children of women with THRT-treated hypothyroidism during pregnancy have developmental outcomes similar to children born to women without prenatal THRT.

Compared with children of mothers who started THRT after delivery, children with prenatal THRT exposure seemed to have a reduced hazard (37% reduction) for language impairment; however, the 95% CI was wide and crossed the null. This hazard reduction was slightly greater in magnitude than the estimated 25% hazard reduction for THRT exposure vs unexposed. This difference may be owing to the low number of diagnosis events in the group of mothers who started THRT after delivery. It is possible that mothers who initiated THRT after delivery might have developed postpartum hypothyroidism due to postpartum thyroiditis, although this possibility cannot be confirmed by the available data. Yet, if it holds true, it is likely that women who initiate THRT after delivery might have had thyroid antibodies already present during pregnancy.^39^ The presence of thyroid antibodies has been previously associated with impaired child development.^40^ In this study, we found no evidence for an association between THRT initiation after delivery and child language and communication skill deficits. However, because children born to women initiating THRT after delivery received a diagnosis of language skill deficits at a younger age than in the other groups, its association with thyroid antibodies during pregnancy needs to be further elucidated.

Habitually low iodine intake is associated with poorer language outcomes.^16^ Insufficient iodine intake reduces the release of unbound thyroxine into the bloodstream, and since only unbound thyroxine crosses the placenta, the fetus might receive too little thyroxine.^41^ However, THRT should increase unbound thyroxine to its reference level, such that the fetus likely receives an adequate supply of unbound thyroxine. This scenario might explain why THRT exposure was not associated with language impairment.

Evidence suggests that maternal thyroid hormone supply is especially crucial during the first weeks of pregnancy.^1^ Parent-reported language outcomes were unchanged when we analyzed the dosage, duration, or timing of exposure via trajectories of gestational THRT.

### Strengths and Limitations

Medical diagnoses have the advantage of representing a clinically relevant threshold of language and speech impairment, and the disadvantage of potentially not capturing more subtle outcomes. In this study, we had the advantage of being able to supplement our analysis with parent-reported language outcomes. However, we cannot exclude some influence of residual confounding because language impairment diagnoses from the NPR were not validated.^42^ The inclusion and analysis of several confounders, including those in the sensitivity analyses, minimizes the risk of bias owing to unmeasured and residual confounding.

The initial low participation rate of 41% is an acknowledged limitation of the MoBa cohort.^6^ Compared with the overall Norwegian population, women in the MoBa study are generally healthier.^8^ However, the percentage of THRT-exposed mother-child pairs was similar to previous prevalence estimates in an unselected sample of the Norwegian population.^43^

Selection bias owing to loss of follow-up was less of an issue in the NPR study sample because this sample did not rely on parents completing follow-up questionnaires after delivery. In the MoBa sample, we cannot exclude such selection bias, which may limit the external validity of the results.^44^ In the NPR sample, the power is sufficient only for large reduction in risks (67% and 86%). In the MoBa sample, we could rule out small mean differences in symptoms.

A prior study, based on the same data, showed that only 50% of women in the THRT-exposed group use THRT consistently.^14^ Therefore, in our study, a significant beneficial effect of THRT on language and communication skills might have been mitigated by mothers who use THRT suboptimally during pregnancy. However, when the analysis was restricted to women who used THRT consistently, no major differences from main results were observed. The risk of bias owing to inconsistent THRT exposure in pregnancy seems therefore to be low in this study.

There is also a possibility that some women in the comparison groups might have had undiagnosed hypothyroidism during pregnancy. Given that the rate of women exposed to THRT during pregnancy in our study is consistent with that in another Norwegian study,^43^ we consider the risk of undiagnosed hypothyroidism to be small and therefore unlikely to be substantially associated with our results.

Because we did not include biological measures of thyroid hormone blood levels, our results may have been biased by confounding due to disease severity. However, when the analysis was restricted to women with a medical diagnosis of hypothyroidism, the effect estimates did not substantially change. Another study found little association with outcomes of adjusting for hormone levels (A.S.F., unpublished data, 2019). An influence on the results might therefore be low.

The reduction in symptoms for pronunciation problems in the complete case analysis is small and likely attributable to selection of healthier women with no missing data on any covariate. Smaller sample sizes in the complete case analysis and the stronger underlying assumptions for unbiased estimates make it likely that this analysis would have yielded biased estimates.^45^

## Conclusions

Although our findings did not reach statistical significance, they demonstrate that children of mothers who receive THRT during pregnancy carry no increased risk for language impairments. Our results support the current recommendations that pregnant women with hypothyroidism should receive THRT.

## Notes

Supplement. **eMethods 1.** Rationale for the Choice of Outcomes **eMethods 2.** Rationale for Sensitivity Analyses **eMethods 3.** Power Calculations **eReferences.** **eTable 1.** Missing-Complete Table of the NPR (N = 53 862) and MoBa (N = 23 686) Study Samples **eTable 2.** Characteristics of MoBa Study Sample (N = 23 686) **eTable 3.** Sensitivity Analyses for Parent-Reported Language and Communication Skill Outcomes Compared With Unexposed Group **eTable 4.** Sensitivity Analyses for Parent-Reported Language and Communication Skill Outcomes Compared With the THRT After Delivery Group **eFigure.** Group-Based Trajectories of THRT Exposure During Gestation (MoBa Study Sample)
