# Supplementary material for: Maternal Thyroid Hormone Replacement Therapy Exposure and Language and Communication Skills of Offspring at 8 Years of Age
Source: JAMA Netw Open. 2019 Oct 2;2(10):e1912424. doi: 10.1001/jamanetworkopen.2019.12424 (PMC6777253; doi:10.1001/jamanetworkopen.2019.12424)
Supplement: Supplement. — eMethods 1. Rationale for the Choice of Outcomes eMethods 2. Rationale for Sensitivity Analyses eMethods 3. Power Calculations eReferences. eTable 1. Missing-Complete Table of the NPR (N = 53 862) and MoBa (N = 23 686) Study Samples eTable 2. Characteristics of MoBa Study Sample (N = 23 686) eTable 3. Sensitivity Analyses for Parent-Reported Language and Communication Skill Outcomes Compared With Unexposed Group eTable 4. Sensitivity Analyses for Parent-Reported Language and Communication Skill Outcomes Compared With the THRT After Delivery Group eFigure. Group-Based Trajectories of THRT Exposure During Gestation (MoBa Study Sample) [file jamanetwopen-2-e1912424-s001.pdf]

Supplementary Online Content

Frank AS, Lupattelli A, Brandlistuen RE, Nordeng H. Maternal thyroid hormone replacement therapy exposure and language and communication skills of offspring at 8 years of age. *JAMA Netw Open.* 2019;2(10):e1912424. doi:10.1001/jamanetworkopen.2019.12424

eMethods 1. Rationale for the Choice of Outcomes
eMethods 2. Rationale for Sensitivity Analyses
eMethods 3. Power Calculations
eReferences.
eTable 1. Missing-Complete Table of the NPR (N=53 862) and MoBa (N=23 686) Study Samples
eTable 2. Characteristics of MoBa Study Sample (N=23 686)
eTable 3. Sensitivity Analyses for Parent-Reported Language and Communication Skill Outcomes Compared With Unexposed Group
eTable 4. Sensitivity Analyses for Parent-Reported Language and Communication Skill Outcomes Compared With the THRT After Delivery Group
eFigure. Group-Based Trajectories of THRT Exposure During Gestation (MoBa Study Sample)

This supplementary material has been provided by the authors to give readers additional information about their work.

© 2019 Frank AS et al. *JAMA Network Open.*

eMethods 1. Rationale for the Choice of Outcomes

Language diagnosis

The rationale for including all diagnoses of language and speech impairments (ICD-10 code: F80) as primary outcome is that we obtain a general picture of the language function. The ICD-code F80 comprises the following sub-diagnoses: F80.0, phonological disorders; F80.1, expressive language disorder; F80.2, mixed receptive-expressive language disorder; F80.4, speech and language development delay due to hearing loss; F80.81, childhood onset fluency disorder; F80.82, Social pragmatic communication disorder; F80.89 Other developmental disorders of speech and language, F80.90, Unspecified, other developmental disorders of speech and language.

Language and communication skill outcomes

The choice of outcomes selected for the MoBa study sample was based on the possibility to detect undiagnosed language problems, as well as on the following rationale (secondary outcomes). We choose items from the Children's Communication checklist (CCC-2) as outcome, because it is the most generally used measure to assess language problems.[1] The Language 20 Semantic Subscale (L-20) is an adaptation to the CCC-2 score for the Norwegian population.[2] Because we work with Norwegian data, we decided to include it into the analysis to capture possible cultural sensitive aspects of language. While CCC-2 is a brief screening instrument to help identify children with potential speech, language and communication needs, L-20 captures the semantic aspects of language. We included the Social Communication Questionnaire (SCQ) questionnaire in order to capture more pragmatic and social communication problems in the children. The SCQ scale has previously been connected with children's verbal Intelligence Quotient.[3] Language aspects connected with phonological problems or difficulties are captured with the Child pronunciation skills questionnaire. We included pronunciation problems because they are part of speech developmental problems and can be co-occurring with other language problems.

© 2019 Frank AS et al. *JAMA Network Open.*

eMethods 2. Rationale for Sensitivity Analyses

Several sensitivity analyses were conducted to assess the robustness of our findings:

We adjusted for paternal confounders (ie, paternal age and educational level), as the father might influence maternal behavior and children's language development.[4]

We adjusted also for the sex of the child to account for potential differences in language development between boys and girls.[5]

The rationale for the complete case analysis was to investigate the effect of missing information on the exposure-outcome associations.

Since the inclusion criteria for the exposure group is having at least one dispensed prescription of thyroid hormones during pregnancy, it is not ascertained whether mothers had transient need for thyroid hormones. Therefore, the rationale for the sensitivity analysis on consistent THRT users was to investigate whether the association of women treated with THRT throughout pregnancy and language development in the offspring is different from the association of women who might not had a transient need for THRT during pregnancy.

In the MoBa sample, maternal depression symptoms at child age of 8 years was added, because it is known that maternal depression symptoms influence children's neurodevelopment.[6]

A family history of reading and writing and language problems, might impact children's language and communication skills at 8 years.[5] To make sure that the results are not influenced by that, we adjusted also for this variable.

Children, which are bilingual, might need longer time to have the same language abilities in the Norwegian language than those who only speak Norwegian at home.[7] This was the rationale for including multilingualism.

To address the potential risk of confounding by indication, we restricted the analysis to women with a hypothyroid diagnosis in NPR or MBRN.

By splitting the THRT exposure groups into disjoint trajectories, we can investigate whether timing, dosage and intensity of THRT use influence the reported symptoms of language development in the children.[8] This analysis could not be performed for the NPR study sample, because there were less than five diagnosis events in the trajectories.

© 2019 Frank AS et al. *JAMA Network Open.*

eMethods 3. Power Calculations

MoBa study sample
We conducted power analysis for a two-group comparison of means, using the two-sided t-test and the Wilcoxon-Mann Whitney (non-parametric, two groups, two-sided) statistical test. We compare the THRT exposed to the population comparison group in order to get an estimate of the minimal detectable effect size (Cohen's d) and calculate sample sizes needed. We used an alpha value of 0.05 and aimed to detect power of 80%. With minimal allocation ratio of 0.023 (532/22,560), we are able to detect a small effect size (d=0.13) for THRT exposed (n=532) and THRT unexposed children (n=22,560). With minimal allocation ratio of 0.89 (532/594), we are able to detect a small effect size (d=0.18) for THRT exposed (n=532) and THRT after delivery (n=594). Power calculation was performed with G*Power.[9]

NPR study sample
We calculate sample sizes needed to perform time-to-event analysis with a Cox-proportional hazard model (at http://www.sample-size.net/sample-size-survival-analysis/).[10] We used an alpha value of 0.05 and aimed to detect power of 80%. For a number of 289 observed events (Table 2) for the THRT exposed and unexposed groups, a 67% reduced risk in the NPR study sample can be detected (with alpha=0.05, beta=0.20, q1=0.023, q2=1-q1, Relative Hazard (RH)=0.33).
For a number of 21 observed events (Table 2) for the THRT exposed and the THRT after delivery group, a 86% reduced risk in the NPR study sample can be detected (with alpha=0.05, beta=0.20, q1=0.89, q2=1-q1, RH=0.14).

© 2019 Frank AS et al. *JAMA Network Open.*

eReferences

1. Timler GR. Use of the Children's communication Checklist—2 for classification of language impairment risk in young school-age children with attention-deficit/hyperactivity disorder. *Am J Speech Lang Pathol.* 2014;23(1):73-83.
2. Ottem E. Tyve spørsmål om språkferdigheter—en analyse om sammenhengen mellom observasjonsdata og testdata [Twenty questions about language performance, Language 20Q]. *Skolepsykologi*. 2009;1:1127.
3. Eaves LC, Wingert HD, Ho HH, Mickelson EC. Screening for autism spectrum disorders with the social communication questionnaire. *J Dev Behav Pediatr.* 2006;27(2 Suppl):S95-S103.
4. Pancsofar N, Vernon-Feagans L. Mother and father language input to young children: Contributions to later language development. *J Appl Dev Psychol.* 2006;27(6):571-587.
5. Snowling MJ, Duff FJ, Nash HM, Hulme C. Language profiles and literacy outcomes of children with resolving, emerging, or persisting language impairments. *J Child Psychol Psychiatr.* 2016;57(12):1360-1369.
6. Deave T, Heron J, Evans J, Emond A. The impact of maternal depression in pregnancy on early child development. *BJOG.* 2008;115(8):1043-1051.
7. Goodz NS. Interactions between parents and children in bilingual families. In: Genesee F, ed. *Educating second language children: the whole child, the whole curriculum, the whole community.* New York, NY: Cambridge University Press; 1994:61-81.
8. Frank AS. Thyroid hormone replacement therapy during pregnancy−Quantifying medication patterns and associated outcomes in the offspring [Doctoral thesis]. Oslo, Norway: Department of Pharmacy, Faculty of Mathematics and Natural Sciences, University of Oslo; 2019.
9. Faul F, Erdfelder E, Buchner A, Lang A-G. Statistical power analyses using G* Power 3.1: Tests for correlation and regression analyses. *Behav Res Methods.* 2009;41(4):1149-1160.
10. Schoenfeld DA. Sample-size formula for the proportional-hazards regression model. *Biometrics.* 1983;39(2):499-503.

© 2019 Frank AS et al. *JAMA Network Open.*

eTable 1. Missing-Complete Table of the NPR (N=53 862) and MoBa (N=23 686) Study Samples

	**NPR study sample (N=53 862)**			**MoBa study sample (N=23 686)**		
**Key variables on complete data**, No. (%)	Complete set, n=40 397 (75.0%)	Missing set, n=13 465 (24.9%)	*P* Value[a]	Complete set, n=13 866 (58.5%)	Missing set, n=9820 (41.5%)	*P* Value[a]
**EXPOSURE**, No. (%)						
THRT exposed, n=1,204/532	881 (2.2)	323 (2.3)	.09	323 (2.3)	209 (2.1)	.225
Unexposed, n=51,282/22,560	38,509 (95.3)	12,773 (94.9)		13,212 (95.3)	9,348 (95.2)	
THRT after delivery, n=1,376/594	1,007 (2.5)	369 (2.7)		331 (2.4)	263 (2.6)	
**OUTCOMES**, No. (%)						
**Diagnosis**						
Language and speech impairment diagnosis (ICD code F-80)	201 (0.5)	99 (0.7)	<.001	Not applicable		
**Parental reports (mean±sd)**						
Communication problems (CCC-2)	Not applicable			−0.060±0.947	0.010±1.022	<.001
Semantic language problems (L-20)				−0.030±0.955	0.018±1.030	<.001
Symptoms of autism (SCQ)				0.029±0.948	−0.013±1.027	.001
Pronunciation problems				−0.025±0.933	0.002±0.982	.028
Speech difficulties				−0.011±0.965	−0.003±0.987	.529
**COVARIATES**, No. (%)						
Maternal age (years)						
≤24	3,790 (9.4)	1,742 (12.9)	<.001	9511 (6.9)	807 (8.2)	<.001
25-29	13,020 (32.2)	4,106 (30.5)		4,419 (31.8)	2,960 (30.1)	
30-34	16,198 (40.1)	4,921 (36.5)		5,780 (41.7)	3,979 (40.5)	
≥35	7,389 (18.3)	2,696 (20.0)		2,716 (19.5)	2,074 (21.1)	
Married/Cohabiting (years)						
Yes	38,977 (96.5)	12,499 (92.8)	<.001	13,483 (97.2)	9,367 (95.4)	<.001
No	1,420 (3.5)	966 (7.2)		383 (2.7)	453 (4.6)	
Parity						
Multiparity	20,911 (51.8)	7,466 (55.4)	<.001	6,991(50.4)	5,380 (54.7)	<.001
Primiparity	19,486 (48.2)	5,999 (44.5)		6,875 (49.6)	4,440 (45.2)	
Mental comorbidity						
Medicated	983 (2.4)	355 (2.6)	.006	292 (2.1)	222 (2.3)	.162
Non-medicated	3,833 (9.5)	1,390 (10.3)		1,242 (8.9)	943 (9.6)	
No	35,581 (88.1)	11,720 (87.0)		12,332 (88.9)	8,655 (88.1)	
Somatic comorbidity						
Medicated	1,664 (4.1)	535 (4.0)	.315	559 (4.0)	374 (3.8)	.009
Non-medicated	2,744 (6.8)	961 (7.1)		1,007 (7.3)	618 (6.3)	
No	35, 989 (89.1)	11,969 (88.9)		12,300 (88.7)	8,828 (89.8)	

© 2019 Frank AS et al. *JAMA Network Open.*

eTable 1. Missing-Complete Table of the NPR (N=53 862) and MoBa (N=23 686) Study Samples (continued)

	NPR study sample (N=53 862)			MoBa study sample (N=23 686)		
**Key variables on complete data**, No. (%)	Complete set, n=40 397 (75.0%)	Missing set, n=13 465 (24.9%)	*P* Value[a]	Complete set, n=13 866 (58.5%)	Missing set, n=9820 (41.5%)	*P* Value[a]
Analgesics						
Yes	17,726 (43.9)	5,220 (38.7)	<.001	5,988 (43.2)	4,202 (42.8)	.545
No	22,671 (56.1)	8,245 (61.2)		7,878 (56.8)	5,618 (57.2)	
Folic acid and other supplements[b]						
Yes	28,465 (70.5)	7,497 (55.7)	<.001	10,228 (73.8)	6,686 (68.1)	<.001
No	11,932 (29.5)	5,968 (44.3)		3,638 (26.2)	3,134 (31.9)	
Fiber intake						
≥29.8 gram/day	19,592 (48.5)	5,278 (39.2)	<.001	6,755 (48.7)	4,808 (48.9)	.710
<29.8 gram/day	20,805 (51.5)	8,187 (60.8)		7,111 (51.3)	5,012 (51.0)	
Protein intake						
≥85.9 gram/day	19,742 (48.9)	5,144 (38.2)	<.001	6,822 (49.2)	4,699 (47.8)	.041
<85.9 gram/day	20,655 (51.1)	8,321 (61.8)		7,044 (50.8)	5,121 (52.2)	
Sex of Child						
Boy	20,722 (51.3)	6,995 (51.9)	.189	7,155 (51.6)	4,995 (50.9)	.265
Girl	19,675 (48.7)	6,470 (48,0)		6,711 (48.4)	4,825 (49,1)	
Major Births Defects						
Yes	1,915 (4.8)	721 (5.3)	<0.001	685 (4.9)	465 (4.7)	.469
No	38,482 (95.2)	12,744 (94.7)		13,181 (95.0)	9,355 (95.3)	
Maternal hypothyroid diagnosis						
Hypothyroidism[c] (ICD-code e03)						
Yes	613 (1.5)	214 (1.6)	.557	222 (1.6)	128 (1.3)	.061
No	39,784 (98.5)	13,251 (98.4)		13,644 (98.4)	9,692 (98.7)	

[a]Differences between groups were calculated with χ^2 test. Significant difference is considered for *P* value <.05.

[b]Other supplement included, vitamins (B, B2, B6, B12, C, D, Niacin, Pantothenic acid, Biotin), Omega 3 fatty acids, and minerals (Calcium, Copper, Chromium, Iodine, Iron, Magnesium, Selenium and Zink)

[c]ICD-10 codes e03 from NPR and MBRN. Thyroid diagnoses are available only for a sub-sample of the study population, because i) reporting thyroid diagnoses is not mandatory in MBRN and ii) information in NPR is incomplete if women got a diagnosis before 2008.

Abbreviations: CCC-2, Children's Communication Checklist, ICD-10, International Classification of Disease code 10th edition, L-20, Language 20 Semantic subscale, MBRN, The Medical Birth Registry of Norway, MoBa, The Norwegian Mother, Father and Child Cohort Study, NPR, The Norwegian Patient Registry, SCQ, Social and Communication Questionnaire, THRT, Thyroid hormone replacement therapy, sd, standard deviation

© 2019 Frank AS et al. *JAMA Network Open.*

eTable 2. Characteristics of MoBa Study Sample[a] (N=23 686)

Variables	Mother-Child Pairs, No. (%)			Standardized Difference of THRT	
	THRT exposed (n=532)	Unexposed (n=22 560)	THRT after delivery (n=594)	Exposed vs unexposed	Exposed vs THRT after delivery
Maternal age (years)					
≤24	22 (4.1)	1,695 (7.5)	41 (6.9)	0.276	0.224
25-29	133 (25.0)	7,069 (31.3)	177 (29.8)		
30-34	217 (40.7)	9,295 (41.2)	247 (41.5)		
≥35	160 (30.1)	4,501 (19.9)	129 (21.7)		
Paternal age (years)					
≤24	11 (2.0)	726 (3.2)	19 (3.2)	0.247	0.167
25-29	83 (15.6)	4,691 (20.8)	110 (18.5)		
30-34	183 (34.4)	8,971 (39.8)	226 (38.0)		
≥35	253 (47.6)	8,109 (35.9)	236 (39.7)		
BMI at conception (kg/m^2)					
≤18	12 (2.2)	646 (2.8)	15 (2.5)	0.278	0.141
19-24	286 (53.7)	14,395 (63.8)	347 (58.4)		
25-29	136 (25.6)	5,271 (23.4)	147 (24.7)		
≥30	86 (16.1)	1,824 (8.1)	69 (11.6)		
Married/Cohabiting					
Yes	510 (95.8)	21,771 (96.5)	569 (95.8)	0.033	0.004
No	22 (4.1)	789 (3.5)	25 (4.2)		
Parity					
Multiparity	310 (58.3)	11,625 (51.5)	320 (53.8)	0.136	0.089
Primiparity	222 (41.7)	10,935 (48.5)	274 (46.2)		
Maternal education (ongoing) in years					
<9	6 (1.1)	167 (0.7)	4 (0.7)	0.058	0.108
9-12	107 (20.1)	4,399 (19.5)	137 (23.0)		
13-16	242 (45.5)	10,099 (44.8)	245 (41.2)		
>16	166 (31.2)	7,529 (33.3)	196 (32.9)		
Paternal education (ongoing) in years					
<9	13 (2.4)	599 (2.6)	27 (4.5)	0.027	0.193
9-12	174 (32.7)	7,356 (32.6)	230 (38.7)		
13-16	154 (28.9)	6,727 (29.8)	150 (25.2)		
>16	163 (30.6)	6,715 (29.7)	155 (26.1)		
Maternal income[b]					
<16,013 USD	109 (20.4)	4,561 (20.2)	135 (22.7)	0.089	0.192
16,013-54,443 USD	311 (58.5)	14,057 (62.3)	375 (63.1)		
>54,443 USD	96 (18.0)	3,409 (15.1)	68 (11.4)		
Smoking during pregnancy					
Yes	18 (3.3)	1,018 (4.5)	43 (7.2)	0.067	0.181
No	445 (83.6)	18,948 (83.9)	477 (80.3)		
Stopped	27 (5.0)	1,351 (5.9)	34 (5.7)		
Alcohol use during pregnancy[c]					
Yes	114 (21.4)	6,021 (26.7)	154 (25.9)	0.152	0.172
No	409 (76.9)	16,055(71.2)	425 (71.5)		
LTHMD					
Yes	189 (35.5)	4,943 (21.9)	177 (29.8)	0.304	0.122
No	336 (63.1)	17,196 (76.2)	406 (68.4)		
Mental comorbidity					
Medicated	24 (4.5)	466 (2.1)	24 (4.0)	0.170	0.044
Non-medicated	63 (11.8)	2,044 (9.1)	78 (13.1)		
No	445 (83.6)	20,055 (88.8)	492 (82.8)		

© 2019 Frank AS et al. *JAMA Network Open.*

eTable 2. Characteristics of MoBa Study Sample[a] (N=23 686) **(continued)**

**Variables**	Mother-Child Pairs, No. (%)			Standardized Difference of THRT	
	THRT exposed (n=532)	Unexposed (n=22 560)	THRT after delivery (n=594)	Exposed vs unexposed	Exposed vs THRT after delivery
Somatic comorbidity[d]					
Medicated	73 (13.7)	825 (3.6)	35 (5.9)	0.400	0.300
Non-medicated	55 (10.3)	1,527 (6.8)	43 (7.2)		
No	404 (75.9)	20,208 (89.6)	516 (86.9)		
Folic acid and other supplements[e]					
Yes	415 (78.0)	16,052 (71.1)	447 (75.3)	0.158	0.065
No	117 (21.9)	6,508 (28.9)	147 (24.7)		
Fiber intake					
≥29.8 gram/day	260 (48.9)	11,019 (48.8)	284 (47.8)	0.001	0.021
<29.8 gram/day	272 (51.1)	11,541 (51.2)	310 (52.2)		
Sex of Child					
Boy	280 (52.6)	11,568 (51.3)	302 (50.8)	0.027	0.036
Girl	252 (47.3)	10,992 (48.7)	292 (49.2)		
Maternal thyroid diagnosis					
Hypothyroidism[f] (ICD-code e03)					
Yes	350 (65.8)	0 (0)	0 (0)	1.945	1.925
No	182 (34.2)	22,560 (100)	594 (100)		
**Additional variables for sensitivity analyses**, No. (%)					
Symptoms of anxiety and depression at child age 8 years[g] (SCL-8)					
Yes	143 (26.9)	5,267 (23.3)	165 (27.8)	0.079	0.025
No	387 (72.7)	17,087 (75.7)	422 (71.0)		
Language spoken at home (MoBa Q5-y)					
Only Norwegian	378 (71.0)	15,229(67.5)	398 (67.0)	0.078	0.124
Norwegian-Other	21 (3.9)	641 (2.8)	15 (2.5)		
Other-Other	0 (0)	22 (0.09)	0 (0)		
Family history of reading and writing difficulties (MoBa Q5-y)					
Not present in family	282 (53.0)	12,324 (54.6)	303 (51.0)	0.108	0.091
Present in one of mother, father or sibling	124 (23.3)	4,491 (19.9)	113 (19.0)		
Present in two of mother, father and sibling	35 (6.5)	1,386 (6.1)	51 (8.6)		
Present in all, mother, father and sibling	20 (3.7)	561 (2.5)	19 (3.2)		

[a]There were in total 41.5 % missing information in important confounders. Missing percentage in variables: Maternal education (n=389 (1.6%)); Income (n=565 (2.4 %)); Alcohol (n=508 (2.1 %)); LTHMD (n=439 (1.8 %)); Smoking (n=1,325 (5.6%)); Education (paternal) (n=1,223 (5.2 %)); Age (paternal) (n=68 (0.3 %)); BMI (n=452 (1.9 %)); Family history of language, reading and writing problems (n=3,977 (16.8%)); Multilingualism at home (n=6,985 (29.5%)); depression symptoms in MoBa Q8-y (n=215 (0.9 %))[b]Women's income status (USD/year): 1.00 NOK ≈ 0.13 USD

[c]Alcohol consumption, No stands for 'less than once a month', and Yes for 'once or more a month'

[d]Somatic comorbidity includes epilepsy, arthritis, anemia, diabetes mellitus (including gestational diabetes mellitus), cardiovascular disorders

[e]Other supplement included, vitamins (B1, B2, B6, B12, C,D, Niacin, Pantothenic acid, Biotin), Omega 3 fatty acids, and minerals (Calcium, Copper, Chromium, Iodine, Iron, Magnesium, Selenium and Zink)

[f]ICD-10 codes e03 from NPR and MBRN. Thyroid diagnoses are available only for a sub-sample of the study population, because i) reporting thyroid diagnoses is not mandatory in MBRN and ii) information in NPR is incomplete if women got a diagnosis before 2008

© 2019 Frank AS et al. *JAMA Network Open.*

[g]Maternal symptoms of anxiety and depression at child age of 8 years was measured by the Hopkins Symptoms Checklist (SCL-8)

Abbreviations: BMI, Body mass index, ICD-10, International Classification of Disease code 10th edition, LTHMD, Lifetime history of major depression, MBRN, The Medical Birth Registry of Norway, MoBa, The Norwegian Mother, Father and Child Cohort Study, MoBa Q5-y, MoBa questionnaire at child age 5 years, NOK, Norwegian Kroner, NPR, The Norwegian Patient Registry, sd, standard deviation, THRT, Thyroid hormone replacement therapy, US, United States of America, USD, US Dollar, vs, versus

© 2019 Frank AS et al. *JAMA Network Open.*

eTable 3. Sensitivity Analyses for Parent-Reported Language and Communication Skill Outcomes Compared With Unexposed Group[a]

**Confounder adjustment and sub-analysis**	Adjusted[b] β (95% CI)				
	CCC−2	L−20	SCQ	Pronunciation problems	Speech difficulty
Parental variables[c] (n=532)	0.02 (−0.07, 0.11)	0.00 (−0.09, 0.09)	0.05 (−0.03, 0.13)	−0.01 (−0.10, 0.08)	0.01 (−0.08, 0.10)
Maternal depression[d] (n=532)	0.02 (−0.07, 0.11)	0.00 (−0.09, 0.09)	0.05 (−0.03, 0.13)	−0.01 (−0.10, 0.08)	0.01 (−0.08, 0.10)
FH of LRW difficulties[e] (n=532)	0.01 (−0.07, 0.10)	0.00 (−0.10, 0.08)	0.05 (−0.03, 0.13)	−0.02 (−0.11, 0.07)	0.01 (−0.09, 0.08)
Multilingualism (n=532)	0.02 (−0.07, 0.10)	0.00 (−0.09, 0.08)	0.05 (−0.03, 0.13)	−0.02 (−0.10, 0.07)	0.01 (−0.08, 0.10)
Sex of child (n=532)	0.01 (−0.07, 0.10)	0.00 (−0.10, 0.08)	0.06 (−0.03, 0.14)	−0.02 (−0.11, 0.07)	0.01 (−0.08, 0.10)
Hypothyroid diagnosis[f] (n=350)	0.00 (−0.10, 0.11)	−0.03 (−0.14, 0.07)	0.03 (−0.08, 0.13)	0.03 (−0.13, 0.07)	−0.02 (−0.12, 0.08)
Complete case analysis (n=13,535)[g]	0.07 (−0.04, 0.18)	−0.01 (−0.12, 0.09)	0.04 (−0.06, 0.14)	−0.07 (−0.15, 0.00)	−0.04 (−0.13, 0.06)
Consistent user (n=399)[h]	0.01 (−0.09, 0.11)	0.01 (−0.09, 0.11)	0.06 (−0.03, 0.16)	0.00 (−0.11, 0.10)	0.01 (−0.09, 0.11)
**Trajectory analysis**	Adjusted[b] β (95% CI)				
Increasing-Medium[i] (n=30)	0.06 (−0.25, 0.37)	0.04 (−0.29, 0.37)	0.07 (−0.25, 0.39)	−0.05 (−0.03, 0.23)	0.02 (−0.30, 0.34)
Constant-Medium[j] (n=184)	0.05 (−0.10, 0.20)	−0.03 (−0.17, 0.10)	0.03 (−0.11, 0.16)	−0.06 (−0.17, 0.05)	0.00 (−0.13, 0.13)
Constant-High[k] (n=318)	0.00 (−0.12, 0.11)	0.01 (−0.11, 0.13)	0.06 (−0.05, 0.17)	0.02 (−0.17, 0.05)	0.02 (−0.10, 0.14)

[a]MoBa sample, n=23,686

[b]Comparison group: **THRT unexposed group** (n=22,560)

[c]Parental variables include, age and educational level

[d]Maternal symptoms of anxiety and depression at child age of 8 years was measured by the Hopkins Symptoms Checklist (SCL−8)

[e]Family history of language, reading and writing difficulties was collected at child age of 5 years from mother, father and siblings

[f]Hypothyroid diagnoses include ICD−10 codes (e03) from MBRN and NPR. Crude estimates (β (95% CI)): CCC−2 (0.02 (−0.08, 0.13)), L−20 ((−0.02 (−0.13, 0.09)), SCQ (0.05 (−0.05, 0.16)), Pronunciation problems (−0.01 (−0.11, 0.08)), Speech difficulty (−0.01 (−0.11, 0.09))

[g] THRT exposed (n=323) and unexposed (n=13,212). Crude estimates (β (95% CI)): CCC−2 (0.09 (−0.02, 0.20)), L−20 (0.00 (−0.11, 0.10)), SCQ (0.05 (−0.05, 0.15)), Pronunciation problems (−0.05 (−0.13, 0.03)), Speech difficulty (−0.03 (−0.12, 0.06))

[h] Crude estimates (β (95% CI)): CCC−2 (0.03 (−0.07, 0.14)), L−20 (0.02 (−0.09, 0.13)), SCQ (0.08 (−0.02, 0.17)), Pronunciation problems (0.01 (−0.09, 0.12)), Speech difficulty (0.02 (−0.08, 0.13))

[i] Crude estimates (β (95% CI)): CCC−2 (0.15 (-0.17, 0.46)), L−20 (0.11 (−0.23, 0.46)), SCQ (0.08 (−0.23, 0.41)), Pronunciation problems (-0.02 (−0.29, 0.26)), Speech difficulty (0.05 (−0.27, 0.38))

[j] Crude estimates (β (95% CI)): CCC−2 (0.07 (−0.08, 0.22)), L−20 (-0.02 (−0.16, 0.12)), SCQ (0.04 (−0.09, 0.17)), Pronunciation problems (-0.05 (−0.16, 0.06)), Speech difficulty (0.01 (−0.12, 0.14))

[k] Crude estimates (β (95% CI)): CCC−2 (0.02 (−0.09, 0.13)), L−20 (0.02 (−0.09, 0.14)), SCQ (0.08 (−0.03, 0.19)), Pronunciation problems (0.03 (−0.09, 0.16)), Speech difficulty (0.02 (−0.09, 0.15))

© 2019 Frank AS et al. *JAMA Network Open.*

Abbreviations: CCC–2, Children's Communication Checklist–2, CI, Confidence Interval, FH, Family history, HR, Hazard ratio, ICD–10, International Classification of Disease code 10th edition, L–20, Language 20 Semantic subscale, LRW, Language, reading and writing, MBRN, The Medical Birth Registry of Norway, MoBa, The Norwegian Mother, Father and Child Cohort Study, NPR, The Norwegian Patient Registry, SCQ, Social and Communication Questionnaire, THRT, Thyroid hormone replacement therapy, β, Standardized mean score difference

© 2019 Frank AS et al. *JAMA Network Open.*

eTable 4. Sensitivity Analyses for Parent-Reported Language and Communication Skill Outcomes Compared With the THRT After Delivery Group[a]

Confounder adjustment and sub-analysis	Adjusted[b] β (95% CI)				
	CCC−2	L−20	SCQ	Pronunciation problems	Speech difficulty
Parental variables[c] (n=532)	0.05 (−0.08, 0.17)	0.03 (−0.09, 0.09)	0.07 (−0.06, 0.19)	−0.05 (−0.18, 0.08)	0.01 (−0.11, 0.14)
Maternal depression[d] (n=532)	0.05 (−0.07, 0.17)	0.04 (−0.08, 0.09)	0.07 (−0.05, 0.19)	−0.04 (−0.18, 0.09)	0.02 (−0.10, 0.15)
FH of LRW difficulties[e] (n=532)	0.04 (−0.08, 0.16)	0.03 (−0.09, 0.08)	0.06 (−0.05, 0.19)	−0.05 (−0.17, 0.08)	0.02 (−0.11, 0.14)
Multilingualism (n=532)	0.04 (−0.08, 0.17)	0.03 (−0.09, 0.08)	0.07 (−0.06, 0.13)	−0.05 (−0.17, 0.08)	0.02 (−0.11, 0.14)
Sex of child (n=532)	0.04 (−0.08, 0.16)	0.03 (−0.09, 0.08)	0.07 (−0.05, 0.19)	−0.05 (−0.18, 0.08)	0.01 (−0.11, 0.14)
Hypothyroid diagnosis[f] (n=350)	0.01 (−0.13, 0.15)	0.00 (−0.14, 0.13)	0.06 (−0.08, 0.19)	−0.07 (−0.20, 0.06)	−0.03 (−0.16, 0.11)
Complete case analysis (n=654)[g]	0.10 (−0.06, 0.27)	0.00 (−0.16,0.17)	0.02 (−0.13, 0.17)	−0.13 (−0.28, 0.01)	−0.04 (−0.19, 0.11)
Consistent user[h] (n=399)	0.04 (−0.04, 0.46)	0.03 (−0.10,0.15)	0.07 (−0.05, 0.21)	−0.03 (−0.17,0.10)	0.02 (−0.12, 0.15)
**Trajectory analysis**	Adjusted[b] β (95% CI)				
Increasing-Medium[i] (n=30)	0.08 (−0.23, 0.39)	0.05 (−0.29, 0.39)	0.09 (−0.26, 0.43)	−0.11 (−0.42, 0.20)	0.01 (−0.34, 0.36)
Constant-Medium[j] (n=184)	0.09 (−0.09, 0.26)	0.00 (−0.16, 0.17)	0.05 (−0.11, 0.22)	−0.08 (−0.23, 0.07)	0.02 (−0.15, 0.18)
Constant-High[k] (n=318)	0.02 (−0.12, 0.16)	0.04 (−0.09, 0.18)	0.08 (−0.06, 0.22)	0.02 (−0.17, 0.13)	0.02 (−0.13, 0.18)

[a]MoBa sample, n=23,686

[b]Comparison group: **THRT after delivery** (n=594)

[c]Parental variables include, age and educational level

[d]Maternal symptoms of anxiety and depression at child age of 8 years was measured by the Hopkins Symptoms Checklist (SCL−8)

[e]Family history of language, reading and writing difficulties was collected at child age of 5 years from mother, father and siblings

[f]Hypothyroid diagnoses include ICD−10 codes (e03) from MBRN and NPR. Crude estimates (β (95% CI)): CCC−2 (0.01 (−0.12, 0.15)), L−20 ((−0.01 (−0.14, 0.13)), SCQ (0.05 (−0.08, 0.18)), Pronunciation problems (−0.05 (−0.19, 0.08)), Speech difficulty (−0.02 (−0.16, 0.12))

[g] THRT exposed (n=323) and THRT after delivery (n=331). Crude estimates (β (95% CI)): CCC−2 (0.08 (−0.07, 0.24)), L−20 (−0.01 (−0.17, 0.15)), SCQ (0.04 (−0.11, 0.19)), Pronunciation (−0.14 (−0.29, 0.01)), Speech problems (0.05 (−0.21, 0.09))

[h] Crude estimates (β (95% CI)): CCC−2 (0.02 (−0.10, 0.16)), L−20 (0.03 (−0.10, 0.17)), SCQ (0.08 (−0.05, 0.20)), Pronunciation (−0.02 (-0.16, 0.11)), Speech problems (0.02 (−0.12, 0.15))

[i] Crude estimates (β (95% CI)): CCC−2 (0.14 (−0.19, 0.46)), L−20 (0.12 (−0.23, 0.48)), SCQ (0.09 (−0.24, 0.43)), Pronunciation (−0.06 (-0.36, 0.24)), Speech problems (0.05 (−0.29, 0.39))

[j] Crude estimates (β (95% CI)): CCC−2 (0.06 (−0.11, 0.23)), L−20 (0.00 (−0.17, 0.16)), SCQ (0.04 (−0.12, 0.20)), Pronunciation (−0.09 (-0.23, 0.05)), Speech problems (0.00 (−0.15, 0.16))

[k] Crude estimates (β (95% CI)): CCC−2 (0.01 (−0.13, 0.15)), L−20 (0.03 (−0.11, 0.18)), SCQ (0.08 (−0.05, 0.22)), Pronunciation (−0.01 (-0.16, 0.15)), Speech problems (0.02 (−0.13, 0.16))

© 2019 Frank AS et al. *JAMA Network Open.*

Abbreviations: CCC−2, Children's Communication Checklist−2, CI, Confidence Interval, FH, Family history, HR, Hazard ratio, ICD−10, International Classification of Disease code 10th edition, L−20, Language 20 Semantic subscale, LRW, Language, reading and writing, MBRN, The Medical Birth Registry of Norway, MoBa, The Norwegian Mother, Father and Child Cohort Study, NPR, The Norwegian Patient Registry, SCQ, Social and Communication Questionnaire, THRT, Thyroid hormone replacement therapy, β, Standardized mean score difference

© 2019 Frank AS et al. *JAMA Network Open.*

eFigure. Group-Based Trajectories of THRT Exposure During Gestation (MoBa Study Sample)

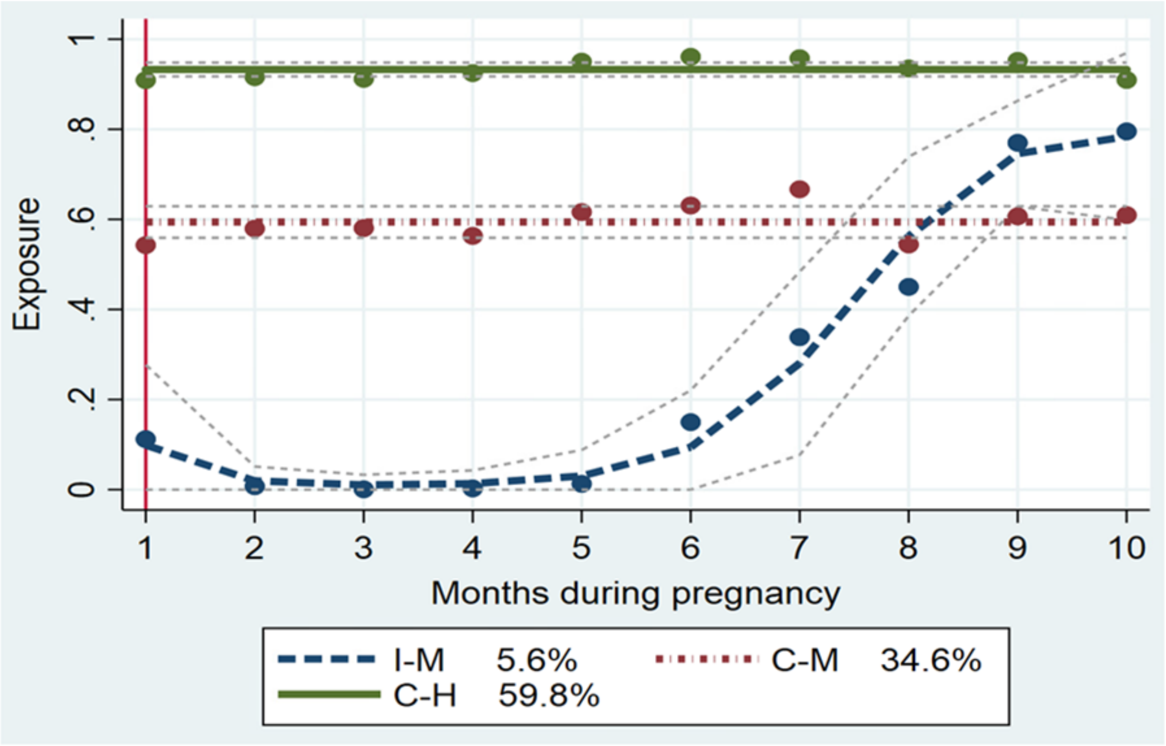

Legend Estimated adherence trajectories (**horizontal lines**), observed group means at each month (**dot symbols**), and estimated percentages for each group: Increasing-Medium (I-M), Constant-Medium (C-M), Constant-High (C-H). **Dashed-thin lines** are approximated 95% pointwise confidence intervals on the estimated trajectories. **Vertical line** marks the start of the gestational period. A month represents 4 gestational weeks.

Abbreviations: I-M, Increasing-Medium trajectory, C-M, Constant-Medium trajectory, C-H, Constant-High trajectory

© 2019 Frank AS et al. *JAMA Network Open.*
